# Clinical validation of a combinatorial PharmAcogeNomic approach in major Depressive disorder: an Observational prospective RAndomized, participant and rater-blinded, controlled trial (PANDORA trial)

**DOI:** 10.1186/s13063-021-05775-8

**Published:** 2021-12-11

**Authors:** Alessandra Minelli, Stefano Barlati, Erika Vitali, Stefano Bignotti, Vincenzo Dattilo, Giovanni Battista Tura, Elisabetta Maffioletti, Edoardo Giacopuzzi, Vincenza Santoro, Giulia Perusi, Chiara Cobelli, Chiara Magri, Silvia Bonizzato, Luisella Bocchio-Chiavetto, Edoardo Spina, Antonio Vita, Massimo Gennarelli

**Affiliations:** 1 Department of Molecular and Translational Medicine, University of Brescia, Brescia, Italy; 2 Genetics Unit, IRCCS Istituto Centro San Giovanni di Dio Fatebenefratelli, Via Pilastroni, 4, 25125 Brescia, Italy; 3 Department of Mental Health and Addiction Services, ASST Spedali Civili of Brescia, Brescia, Italy; 4 Department of Clinical and Experimental Sciences, University of Brescia, Brescia, Italy; 5 Psychiatry Unit, IRCCS Istituto Centro San Giovanni di Dio Fatebenefratelli, Brescia, Italy; 6 Clinical and Experimental Medicine, University of Messina, Messina, Italy; 7 Faculty of Psychology, eCampus University, Novedrate, Como, Italy

**Keywords:** Major depressive disorder, Depression, Pharmacogenetic testing, Randomized controlled clinical, Precision medicine, Antidepressant response, Efficacy

## Abstract

**Background:**

Major depressive disorder (MDD) is a common, chronic, debilitating mood disorder that causes serious functional impairment and significantly decreased quality of life. Pharmacotherapy represents the first-line treatment option; however, only approximately one third of patients respond to the first treatment because of the ineffectiveness or side effects of antidepressants. Precision medicine in psychiatry might offer clinicians the possibility to tailor treatment according to the best possible evidence of efficacy and tolerability for each subject. In this context, our study aims to carry out a clinical validation of a combinatorial pharmacogenomics (PGx) test in an Italian MDD patient cohort with advocacy license independence.

**Methods:**

Our study is a prospective participant- and rater-blinded, randomized, controlled clinical observational trial enrolling 300 MDD patients who are referred to psychiatric services to receive a new antidepressant due to the failure of their current treatment and/or the onset of adverse effects. Eligible participants are randomized to the TGTG group (Treated with Genetic Test Guide) or TAU group (Treated as Usual). For all subjects, DNA is collected with a buccal brush. The primary outcome is the reduction in depressive symptomatology. The secondary outcomes involve a range of scales that assess MDD symptoms and social functioning outcomes. The assessment is performed at four timepoints: baseline and 4, 8, and 12 weeks.

**Discussion:**

This project represents the first randomized controlled clinical trial to investigate whether a non-commercial PGx test improves outcomes in an MDD naturalistic cohort. Moreover, the identification of new genetic variants associated with non-response or side effects will improve the efficacy of the test, leading to further cost-saving.

**Trial registration number:**

ClinicalTrials.gov NCT04615234. Registered on November 4, 2020.

**Supplementary Information:**

The online version contains supplementary material available at 10.1186/s13063-021-05775-8.

## Background

Major depressive disorder (MDD) is the most common psychiatric disease worldwide and represents a leading cause of years lived with disability, resulting in a substantial socio-economic impact [1]. The main goal of treating MDD is to achieve remission and to maintain the therapeutic effects over time. Despite the availability of different classes of antidepressant drugs (ADs), the success of pharmacological treatment is still unsatisfactory, and matching a patient to his/her optimal treatment generally requires multiple trials of different treatments, with the sobering observation that the more treatments that are tried without success, the less likely a successful outcome is. Only approximately one third of patients achieve remission after the first treatment course, while another approximately one third develop treatment-resistant depression (TRD) [2, 3]. The high percentage of treatment failure or incomplete remission could be a consequence of intrinsic biological and environmental heterogeneity among MDD patients [4, 5], suggesting that biomarkers of the response to ADs would be useful for clinicians to guide treatment at the individual level. In this context, pharmacogenomics (PGx) testing has the potential to reduce antidepressant discontinuation due to side effects and increase efficacy.

Recently, assay-guided treatment has shown promising results. Several observational and randomized controlled trials (RCTs) have been conducted to investigate the impact of pharmacogenetics or pharmacogenomics testing on antidepressant outcome in MDD patients with interesting results [6, 7].

This article presents the protocol for the RCT and is written to comply with the recommended SPIRIT guidelines for RCT protocols (Additional file 1).

### Aim of the study

**Fig. 1:**
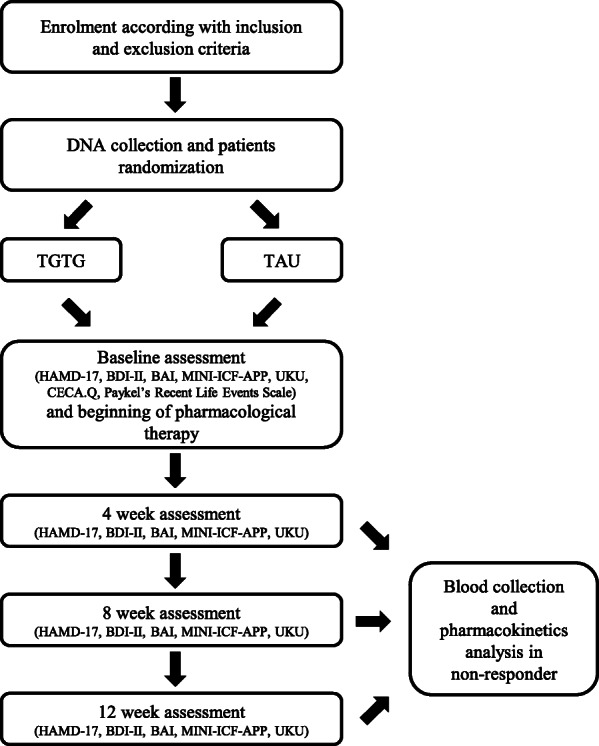
Flowchart of the trial design

**Fig. 2:**
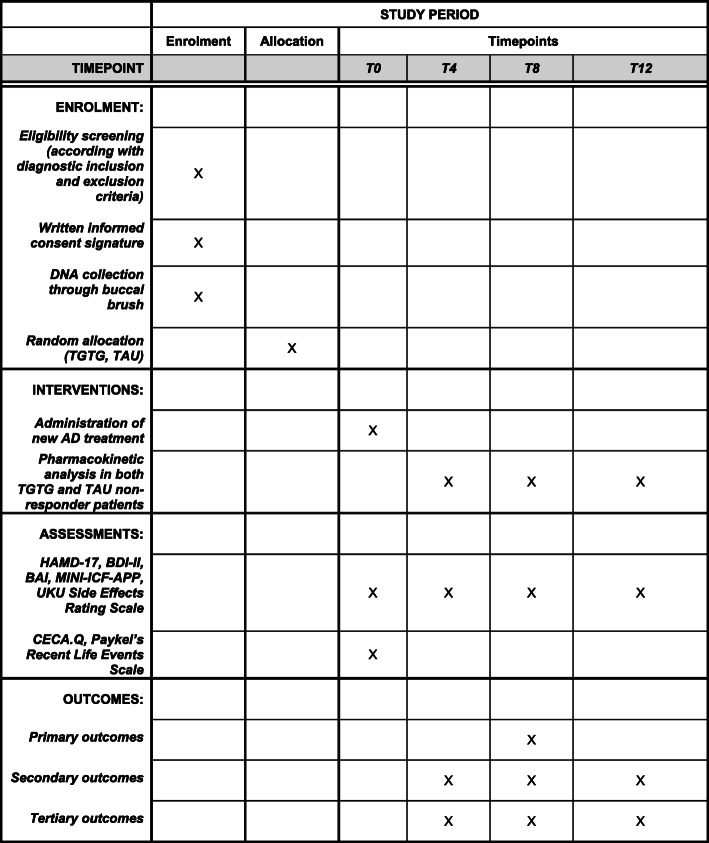
Schedule of enrolment, interventions, assessments, and outcomes of the PANDORA trial

The study was designed as an observational, prospective participant- and rater-blinded randomized, controlled trial (shown in Figs. 1 and 2) to evaluate the clinical efficacy of a combinatorial PGx test to guide clinician’ treatment decision-making in a naturalistic setting. This study will be conducted in an Italian MDD patient cohort with advocacy license independence. In particular, the main objective of the study is to assess the role of a PGx test in improving the response rate and leading to a greater amelioration of depressive symptoms in MDD patients. The secondary objective is to evaluate the use of PGx in decreasing the side effects of antidepressants. Moreover, the study aims to provide data about the use of the PGx test in decreasing depressive-related symptomatology (such as anxiety symptoms and functionality) and the possible influence of vulnerability factors, such as early and recent stressful events, on the main outcomes.

## Methods

### Eligibility criteria

Three hundred MDD out-patients are voluntarily enrolling in the study. Patients are referred to psychiatric services (University Department of Mental Health, Spedali Civili Hospital, and IRCCS Istituto Centro San Giovanni di Dio Fatebenefratelli – Brescia, Italy) to receive a new AD due to the failure of their current treatment and/or the onset of adverse effects. The diagnostic inclusion criteria are as follows: a current diagnosis of unipolar depression according to the Diagnostic and Statistical Manual of Mental Disorders-5 (DSM-5) [8] classification system criteria with a Hamilton Depression Rating Scale (HAMD-17) [9] score ≥14, age range 18–65 years, and Caucasian ethnicity. The exclusion criteria are cognitive impairment (Mini Mental State Examination MMSE <24) [10]; neurological disorders; diagnosis of MDD with psychotic features, bipolar I and II disorders, schizophrenia spectrum and other psychotic disorders, obsessive-compulsive disorder, post-traumatic stress disorder, alcohol and substance abuse in the last 3 months; comorbidity with personality disorders (cluster A and/or B); pregnancy; and comorbidity with other severe medical illness. Diagnoses are confirmed using the Italian version of the Structural Clinical Interview for DSM-5 disorders (SCID-5-CV) [11] and the Structural Clinical Interview for personality disorders (SCID-5-PD) [12]. After the enrolment of MDD patients in accordance with the inclusion and exclusion criteria and after obtaining written informed consent, for all patients, DNA is collected through a buccal brush.

### Randomization and concealment

Eligible patients are randomized to the Treated with Genetic Test Guide (TGTG) group or Treated as Usual (TAU) group. The allocation to the TGTG or TAU group is performed with simple randomization software (http://glimmpse.samplesizeshop.org/) by the project manager who is not involved in either the evaluation or the treatment. The allocation of the patients is communicated directly from the project manager (AM) to the prescriber. None of the other people involved in the projects are informed and/or have access to the allocation information. The randomized allocation sequence of the trial participants is conserved in a double file protected by a double password owned by the PI (MG) and the project manager (AM).

### Assessments

The clinical assessment is carried out at 4 timepoints: baseline (T0), after 4 (T4), 8 (T8), and 12 (T12) weeks of AD treatment. The evaluations are performed using the HAMD-17, the Beck Depression Inventory II (BDI-II) [13]; the Beck Anxiety Inventory (BAI) [14], to evaluate the clinical efficacy of the therapy; the MINI-ICF-APP [15] to monitor changes in psychosocial functioning; and the UKU (Udvalg for Kliniske Undersogelser) Side Effects Rating Scale [16] to observe the adverse events. Moreover, the Childhood Experience of Care and Abuse Questionnaire (CECA.Q) [17] and the Paykel’s Recent Life Events Scale [18] are assessed only at T0.

### Blinding

All the participants are evaluated by assessors who are not otherwise involved, pharmacological treatment, or patient allocation. The treating physicians are unblinded. Neither the assessors nor prescribers are involved in the data analysis.

### Pharmacogenetic report and intervention procedure

The clinicians of the patients in the TGTG group receive the PGx test report within 48 h, and all the participants start their new treatment within 72 h. The PGx test results for the subjects in the TAU are provided to the prescriber once all week 12 visit procedures are completed. AD monotherapy is mandatory, with the exception of the association of benzodiazepines and/or hypnotics when necessary. If the patients need another change in antidepressant monotherapy, they stay in the study protocol, and the changes are annotated. If the patients need a combination and/or augmentation treatment with antipsychotics and/or mood stabilizers, they are excluded from the study.

### Outcomes

The primary outcome is symptom improvement at week 8, as measured by the percent change in the HAMD-17 score from baseline, between the two groups.

Secondary outcomes include response and remission rates at 4, 8, and 12 weeks according to the HAMD-17.

Tertiary outcomes include (1) changes in scores of depressive symptoms at 4, 8, and 12 weeks compared with baseline according to the BDI-II; (2) response and remission rate at 4, 8, and 12 weeks according to BDI-II; (3) changes in scores of anxiety symptoms at 4, 8, and 12 weeks compared with baseline as measured by the BAI; (4) changes in scores of psychosocial functioning at 8 and 12 weeks compared with baseline as measured by the MINI-ICF-APP; and (5) side effects at 4, 8, and 12 weeks as assessed by the UKU Side Effect Rating Scale.

The response is defined as a ≥50% decrease in the assessment of interest (HAMD-17, BDI-II) at weeks 4, 8, and 12 compared with the baseline. Remission is defined as a score of ≤7 for HAMD-17 and ≤9 for BDI-II.

### Genotyping

Buccal cell samples are collected by FLOQSwab hDNA Free buccal brushes (Copan Brescia, Italy). Genomic DNA extraction is performed with a Quick DNA Miniprep plus Kit (ZymoResearch, California, USA) according to the manufacturer’s instructions.

In our trial, we investigate the genetic variants reported in the PharmGKB database (www.pharmgkb.org) with a clinical annotation of evidence of association with AD response classified as levels 1a, 1b, 2a, and 2b.

Our PGx test includes 31 genetic variants. Thirty single-nucleotide polymorphisms (SNPs) are genotyped with customized TaqMan OpenArray plates on a QuantStudio 12K Flex Real-Time PCR System (Applied Biosystems, Foster City, California, USA) according to the manufacturer’s instructions (15 in *CYP2D6*, 10 in *CYP2C19*, and four in *MC4R*, *FKBP5*, *HTR1A*, and *HTR2A*). The data are analyzed with the Genotyping application on Thermo Fisher Cloud. Moreover, copy number variation (CNV) of the *CYP2D6* gene is evaluated using the TaqMan Copy Number Assay mix specific for *CYP2D6* exon 9 (Assay ID: Hs00010001_cn) according to the manufacturer’s instructions and with a C_T_ threshold of 0.2, and the analysis is performed with CopyCaller Software (Applied Biosystems). Using AlleleTyper Software (Life Technologies, California, USA), we integrate the SNP genotyping results with the copy number information for the *CYP2D6* gene to obtain all the eventual *CYP2D6* and *CYP2C19* diplotypes. Diplotypes with higher allele frequencies in the European population are selected if it cannot be determined with certainty. The translation is based on the translation table obtained from the PharmGKB database.

Participants are also genotyped for the 5-HTTLPR (short/long allele) in the SLC6A4 gene by PCR amplification of the relevant genomic location using the KAPA HiFi HotStart PCR Kit (Roche Diagnostics, Basel, Switzerland) and the following primers: 5′–ATGCCAGCACCTAACCCCTAATGT–3′ (forward) and 5′–GGACCGCAAGGTGGGCGGGA–3′ (reverse). The genotype is then determined by electrophoresis and visualization of the amplified products on a 2% agarose gel.

### Pharmacogenomic (PGx) test report

Considering the resulting genetic profile, a personalized PGx report is generated. The diplotypes obtained for the *CYP2D6* and *CYP2C19* genes are associated with the matching metabolizer phenotypes: ultra-rapid, normal, intermediate, and poor for *CYP2D6*, and ultra-rapid, rapid, intermediate, poor, normal, likely intermediate, and likely poor for *CYP2C19.*

**Fig. 3:**
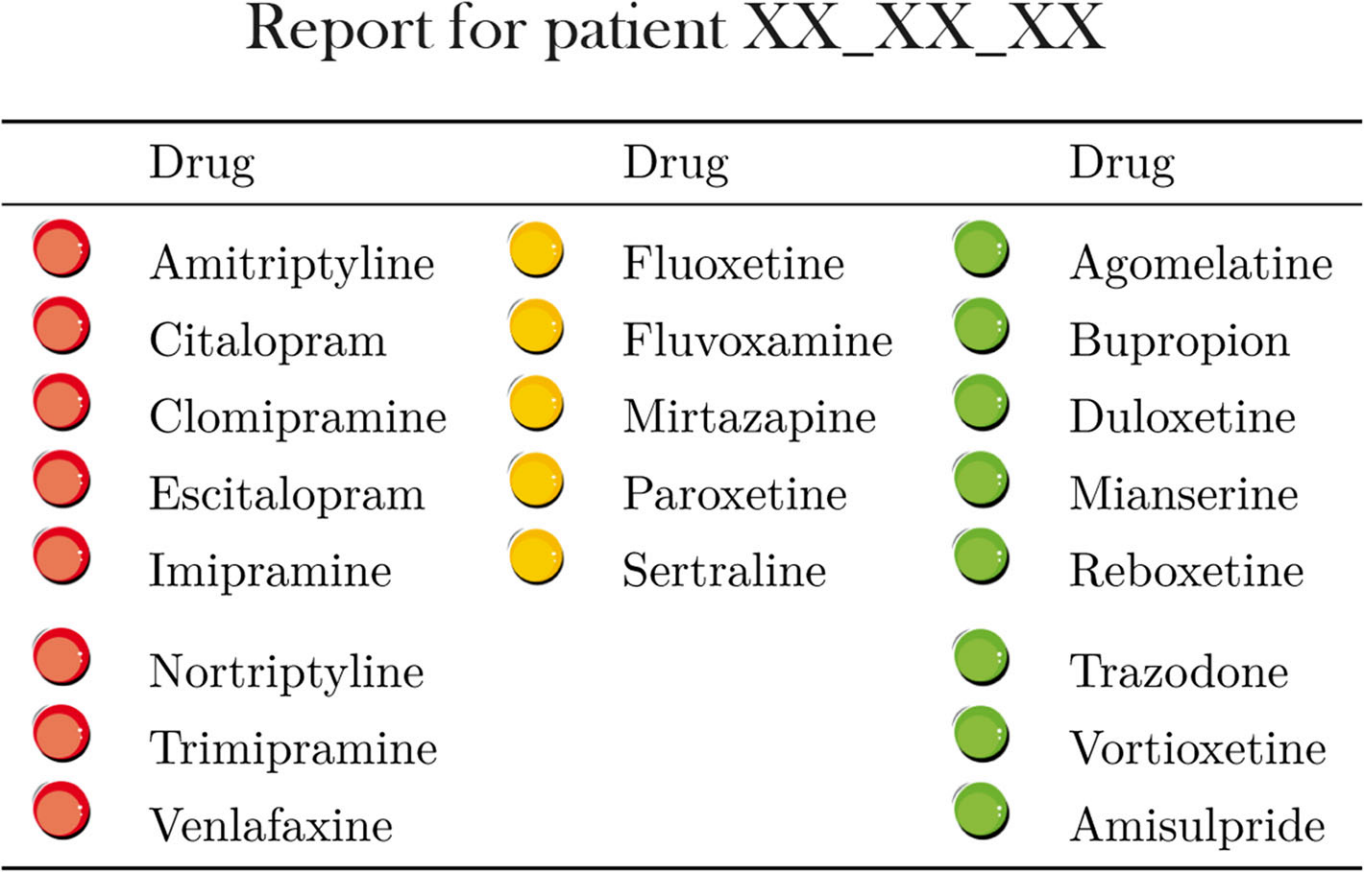
Example of a report. The report shows the classification of drugs in the three recommended categories, green (“use as directed”), yellow (“use with caution”), and red (“use with extreme caution”). The current AD (“1st drug”) is excluded from the list to avoid clinician bias in decision-making

According to both the Clinical Pharmacogenetics Implementation Consortium (CPIC) and the Dutch Pharmacogenetics Working Group (DPWG) guidelines, the report places the most ADs widely used in Italy into three recommended categories: (1) “use as directed” (labeled “green”), (2) “use with caution” (labeled “yellow”), and (3) “use with extreme caution” (labeled “red”) (see Fig. 3 for an example of a report for one patient). In addition, drug details associated with each medication in the yellow or red categories are provided. The current AD is excluded from the report to avoid clinician bias in decision-making. The clinicians have access to the PGx report through an interactive web interface.

Before study initiation, training was provided to all participating investigators on the interpretation of genetic testing results and on the relevance of each genetic variant to pharmacotherapy.

### Pharmacokinetic analysis

Pharmacokinetic evaluation is conducted at weeks 4, 8, and 12 in the TAU group and the non-responder patients in the TGTG group. Serum concentrations of the ADs and their metabolites are measured at a steady-state on the same day of clinical evaluation. Blood samples for pharmacokinetic analysis are drawn at 8 a.m. before the morning AD dose. Determination of the serum concentrations of the ADs and their metabolites are performed by using high-performance liquid chromatography procedures.

### Further pharmacogenomics analysis

To better elucidate the molecular mechanisms underlying the response to the ADs, for all non-responder patients in our trial, further PGx analysis will be carried out. In particular, we will first investigate, always through a TaqMan OpenArray approach, further genetic variants known to be involved in the response to ADs or their side effects that are already described in literature studies and in the PharmGKB database. Moreover, for those patients (belonging to both the TGTG and TAU groups) who do not benefit from the assigned treatment, sequencing of all the genes associated with both AD response and susceptibility to side effects will be conducted with the aim of identifying novel rare functional variants.

### Sample size

Based on previous PGx studies performed on MDD patient cohorts [19, 20], the sample size needed to detect a significant reduction in symptom scores was calculated. The analysis revealed that a sample size of 225 participants (⁓112 per group) is required to observe a 15% reduction in symptom scores with a common standard deviation of 45%, alpha = 0.05, and power = 80%. Assuming an estimated drop-out rate of 20–25%, as already observed in follow-up studies on MDD [21], a total sample size of 300 participants will be enrolled in this study.

### Data management

The data management process is the responsibility of the project coordinator. Both clinical and biological data collection, analysis, storage, security, and sharing are consistent with the standard operating procedures that ensure patient pseudonymization.

### Baseline demographic and clinical features

Age (years) and mean (SD); gender (%F); education (years) and mean (SD); race (%); smokers (%); body mass index (BMI) and mean (SD); age of onset (years) and mean (SD); depression category; moderate, severe, or very severe (%); recurrence (%); presence of psychotic symptoms (%); comorbidity with personality disorders congruent with inclusion/exclusion criteria (%); comorbidity with anxiety disorders (%); presence of psychiatric disorders among the first-degree relatives (%); HAMD-17 at baseline and mean (SD); BDI-II at baseline and mean (SD); BAI-II at baseline and mean (SD); mini-ICF-APP at baseline and mean (SD); MMSE at baseline and mean (SD); UKU at baseline and mean (SD); previous failed adequate treatment; CECA-Q mother antipathy (hostility, coldness) (%); CECA-Q father antipathy (hostility, coldness) (%); CECA-Q mother neglect (%); CECA-Q father neglect (%); CECA-Q physical abuse mother (%); CECA-Q physical abuse father (%); CECA-Q sexual abuse (%); and Paykel score and means (SD).

### Statistical analysis

Analysis of variance (ANOVA) or the Kruskal–Wallis and Mann–Whitney *U* nonparametric tests and the chi-squared test will be used to analyze differences in continuous and categorical variables, respectively, between groups. Pearson’s correlation analysis will be used to evaluate bivariate correlations. Parametric and nonparametric tests will be used to meet relative assumptions (i.e., distribution, sample size).

To pursue our primary outcome, the analyses will be performed for patients who complete the study through week 8. The analysis will be performed using mixed-effects models with repeated measures (MMRM) to examine the effect of time and group (TGTG vs. TAU) on the reduction in the HAMD-17 score. The model includes the fixed effect continuous factor baseline HAMD-17 and fixed effect categorical factors, which are the treatment group (TGTG and TAU; 2 levels), visit (weeks 4 and 8; 2 levels), and treatment x visit interaction. The mean changes in the HAMD-17 at week 8 in the TGTG and TAU groups will be estimated and tested utilizing the LS means from the treatment x visit interaction in the MMRM model. The primary analysis will test the difference (contrast) between the week 8 least squares (LS) means at a two-sided significance of 0.05. To achieve the secondary outcome, the generalized linear mixed model will be used for response and remission analyses. The analyses of the tertiary outcomes will be performed for exploratory purposes with the same statistical models. Comparisons between the TGTG and TAU means at all timepoints of evaluation will also be performed for descriptive purposes. Finally, an intention-to-treat (ITT) analysis will be performed for all the patients who undergo at least one post-treatment assessment for efficacy during the study. The last observation will be carried forward on the HAMD-17. All the statistical analyses will be performed using R Statistical Software (R Foundation for Statistical Computing, Vienna, Austria).

## Discussion

Genetic variants play important roles in the responses of MDD patients to ADs, explaining approximately 40% of pharmacological treatment outcomes [22, 23]. Based on this evidence, different studies have been performed to evaluate the utility of treatment guided by a PGx test, which investigates the possible response to ADs according to the genetic background of the patient. These studies revealed a better outcome in patients treated with guided care, both in terms of response rates and in terms of a decrease in reported side effects, confirming the utility of the PGx test in the treatment of MDD [24]. However, few of these studies performed are RCTs, and consequently, further ones are needed to increase the understanding regarding the clinical utility of such tests that include both genetic profile characterization and clinical assessment symptomatology.

In this context, the aim of our study is to evaluate the clinical utility of a combinatorial PGx test by performing an observational, prospective, participant -and rater-blinded, randomized, controlled trial in an Italian MDD patient cohort with advocacy license independence.

The use of a tool based on a combinatorial approach provides clinicians with more complete information about a patient’s response to drugs. Indeed, although individual gene test panels provide information about the effects of an individual gene on each investigated drug, the combinatorial PGx test considers the simultaneous effects of different genes on drug pharmacokinetics and pharmacodynamics, providing information that is more accurate and rapidly applicable to clinician practice [25].

The longitudinal evaluation of outcomes at four different timepoints allows us to assess the efficacy of the PGx test to suggest a therapy that could be efficient in the long term. Moreover, the application of a wide range of rating scales provides a complete view of outcomes, both in terms of symptom improvement and the development of adverse effects, and allows us to study the impact of the PGx test on the different symptom phenotypes of the disease. Moreover, the high number of variants investigated along with a wide range of clinical symptoms characterization that will be performed, allowing us to evaluate the possible association between endophenotypes and specific symptom improvement.

In addition, this trial will provide further information about the genetic variance and the distribution of phenotype metabolizers in an Italian sample of patients with MDD, increasing the amount of data available for the scientific community. Furthermore, in non-responder patients, an in-depth investigation of further genetic variants implicated in treatment outcomes will contribute to enriching the knowledge about the molecular mechanisms underlying the response to ADs.

Finally, the periodical update of the PGx report software will allow us to provide indications for each AD based on more recent versions of the CPIC and DPWG guidelines.

There are some possible limitations of this study. First, as in all the other PG test validation studies, the treating clinician is not blinded to the study arm. This is necessary for ethical issues related to mandating prescribed medications to blind clinicians. To mitigate this limitation, raters and patients were blinded to the study arm until week 12. Second, the majority of the cohort that will be recruited will be limited to the psychiatric services of the Lombardy region. This could affect the project both in terms of impact on the disease and treatment outcome due to the local organization of mental health services and in terms of ethnicities represented in the cohort. This may limit the generalizability of the results to a wider population of MDD patients.

### Trial status

This article is based on the study protocol version 1.2 of August 8, 2018. The study protocol was posted to ClinicalTrials.gov as NCT04615234 (Registration date: November 4, 2020). The recruitment of patients started on February 1, 2020, and will be completed after approximately 30 months. Due to the COVID-19 emergency, the period required for the completion of the study will be longer.

## Supplementary Information

**Additional file 1.** SPIRIT 2013 Checklist: Recommended items to address in a clinical trial protocol and related documents*.

## Data Availability

For processing the data, we use the pseudonymization procedure so that the same data can no longer be attributed to a specific subject without the use of additional information. The correspondence between the code and the personal information of the subject is contained in a separate file protected by passwords. Only the PI (MG) and the project manager (AM) have access to the final dataset. Patient data as well as the statistical analyses are stored on a local server of the IRCCS Istituto Centro San Giovanni di Dio Fatebenefratelli respective international data security standards.

## Abbreviations

MDD: Major depressive disorder
ADs: Antidepressant drugs
TRD: Treatment-resistant depression
PGx: Pharmacogenomics
RCTs: Randomized controlled trials
DSM-5: Diagnostic and Statistical Manual of Mental Disorders-5
HAMD-17: Hamilton Depression Rating Scale
MMSE: Mini Mental State Examination
SCID-5-CV: Structural Clinical Interview for DSM-5 disorders
SCID-5-PD: Structural Clinical Interview for personality disorders
TGTG: Treated with Genetic Test Guide
TAU: Treated As Usual
BDI-II: Beck Depression Inventory II
BAI: Beck Anxiety Inventory
UKU: Udvalg for Kliniske Undersogelser
CECA.Q: Childhood Experience of Care and Abuse Questionnaire
PharmGKB: Pharmacogenomics Knowledge Base
SNPs: Single-nucleotide polymorphisms
CNV: Copy number variation
CPIC: Clinical Pharmacogenetics Implementation Consortium
DPWG: Dutch Pharmacogenetics Working Group
